# Thermophiles; or, the Modern Prometheus: The Importance of Extreme Microorganisms for Understanding and Applying Extracellular Electron Transfer

**DOI:** 10.3389/fmicb.2019.00818

**Published:** 2019-04-26

**Authors:** Bradley G. Lusk

**Affiliations:** ScienceTheEarth, Mesa, AZ, United States

**Keywords:** extremophile, thermophile, extracellular electron transfer, bioelectrochemical system, Gram-positive, biotechnology, biogeochemistry, astrobiology

## Abstract

Approximately four billion years ago, the first microorganisms to thrive on earth were anaerobic chemoautotrophic thermophiles, a specific group of extremophiles that survive and operate at temperatures ∼50 – 125°C and do not use molecular oxygen (O_2_) for respiration. Instead, these microorganisms performed respiration via dissimilatory metal reduction by transferring their electrons extracellularly to insoluble electron acceptors. Genetic evidence suggests that Gram-positive thermophilic bacteria capable of extracellular electron transfer (EET) are positioned close to the root of the Bacteria kingdom on the tree of life. On the contrary, EET in Gram-negative mesophilic bacteria is a relatively new phenomenon that is evolutionarily distinct from Gram-positive bacteria. This suggests that EET evolved separately in Gram-positive thermophiles and Gram-negative mesophiles, and that EET in these bacterial types is a result of a convergent evolutionary process leading to homoplasy. Thus, the study of dissimilatory metal reducing thermophiles provides a glimpse into some of Earth’s earliest forms of respiration. This will provide new insights for understanding biogeochemistry and the development of early Earth in addition to providing unique avenues for exploration and discovery in astrobiology. Lastly, the physiological composition of Gram-positive thermophiles, coupled with the kinetic and thermodynamic consequences of surviving at elevated temperatures, makes them ideal candidates for developing new mathematical models and designing innovative next-generation biotechnologies.

KEY CONCEPTS

**Anaerobe:** organism that does not require oxygen for growth.

**Chemoautotroph:** organism that obtains energy by oxidizing inorganic electron donors.

**Convergent Evolution:** process in which organisms which are not closely related independently evolve similar traits due to adapting to similar ecological niches and/or environments.

**Dissimilatory Metal Reduction:** reduction of a metal or metalloid that uses electrons from oxidized organic or inorganic electron donors.

**Exoelectrogen:** microorganism that performs dissimilatory metal reduction via extracellular electron transfer.

**Extremophiles:** organisms that thrive in physical or geochemical conditions that are considered detrimental to most life on Earth.

**Homoplasy:** a character shared by a set of species that is not shared by a common ancestor

**Non-synonymous Substitutions (K_*a*_):** a substitution of a nucleotide that changes a codon sequence resulting in a change in the amino acid sequence of a protein.

**Synonymous Substitutions (K_*s*_):** a substitution of a nucleotide that may change a codon sequence, but results in no change in the amino acid sequence of a protein.

**Thermophiles:** a specific group of extremophiles that survive and operate at temperatures ∼50–125°C.

## Perhaps You’d Better Start from the Beginning

Approximately four billion years ago (Ga), the first microorganisms to thrive on earth were likely anaerobic chemoautotrophic thermophiles, a specific group of extremophiles that survive and operate at temperatures ∼50–125°C and do not use molecular oxygen (O_2_) for respiration (Pace, 1991; Kashefi and Lovley, 2003; Seckbach, 2004; Sleep, 2018); although this is under investigation (Islas et al., 2003; Boussau and Gouy, 2006; Glansdorff et al., 2008; Weiss et al., 2018). These early microorganisms lived underwater near hydrothermal vents- where modern thermophiles persist (Seckbach, 2004, 2006; Slobodkin et al., 2006; Onyenwoke et al., 2007; Zavarzina et al., 2007; Niu et al., 2009; Slepova et al., 2009). These locations were ideal since they protected microorganisms from ultraviolet radiation and contained plentiful amounts of oxidized compounds including SO_4_^-^, Fe(III) oxides, NO_3_^-^, and Mn(IV) that were used as terminal electron acceptors (Lovley, 1993a; Nealson and Conrad, 1999; Amend and Shock, 2001; Knoll, 2003; Slobodkin, 2005; Seckbach, 2006). Since many of these minerals are insoluble, microorganisms, or exoelectrogens, adapted mechanisms to perform EET for their survival. Positioned close to the root of the Bacteria kingdom on the tree of life, thermophiles provide a glimpse into some of Earth’s earliest forms of respiration via dissimilatory metal reduction (Ciccarelli et al., 2006; Seckbach, 2006; Logan, 2009).

Within the kingdom Bacteria, there are two distinct classifications – Gram-positive and Gram-negative – that span across many bacterial phyla (Ventura et al., 2007; Vesth et al., 2013). This classification has profound implications on the physiology and structure of the bacterial cell, specifically the structure of the cell wall and membrane. This is significant because exoelectrogens are required to transfer their electrons externally through their cell membranes (Lovley, 1993b; Bond and Lovley, 2003; Leang et al., 2003; Lies et al., 2005; Richter et al., 2008; Coursolle et al., 2010; Holmes et al., 2016).

In Gram-negative bacteria, the cell contains two membranes, an inner membrane and an outer membrane, that are separated by a periplasm. Within the periplasmic space is a thin layer (∼5–10 nm) of peptidoglycan/murein, which is a polymer of sugars, NAG, NAM, and amino acids that accounts for approximately 10% of the dry weight of the cell (Koch, 2006). For EET to occur, electrons must traverse a series of peripheral and integral proteins and cytochromes that are imbedded in the inner membrane, span across the periplasm, and are docked to the outer membrane (Bird et al., 2011).

In contrast, Gram-positive bacteria have a cell wall composed of a single membrane and a relatively thick (20–80 nm) peptidoglycan layer which are separated by a periplasmic space. The peptidoglycan consists of many layers and can weigh as much as 60% of the cell’s total dry weight (Koch, 2006; Ehrlich, 2008; Pham et al., 2008,). In addition, Gram-positive bacteria have teichoic acids that have been implicated as the metal binding sites for the cells, are embedded in the cell wall, and extend from the cell membrane to the outer surface of the peptidoglycan layer (Beveridge and Murray, 1980; Beveridge et al., 1982; Ehrlich, 2008). The presence of a thick peptidoglycan layer, sometimes surrounded by an S-layer, makes it necessary for many metal reducing Gram-positive bacteria to conduct EET via proteins and cytochromes that are packed into fissures within the cell wall, anchored to the peptidoglycan, or positioned along teichoic acids (Ehrlich, 2008; Carlson et al., 2012).

**FIGURE 1:**
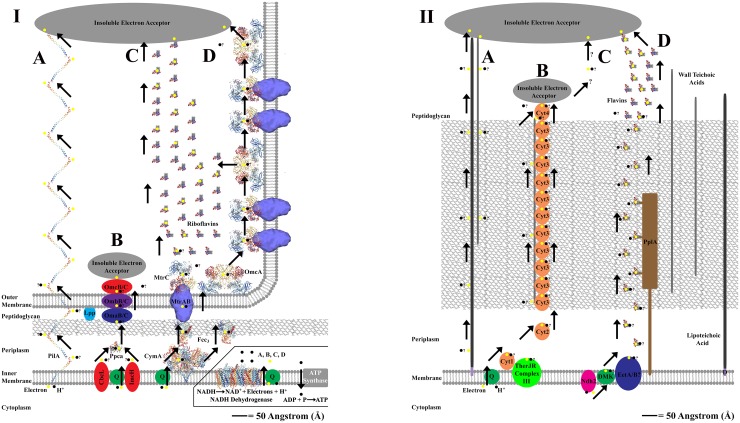
Panel **(I,II)** shows proposed extracellular electron transfer (EET) mechanisms for Gram-negative and Gram-positive bacteria. An angstrom scale is used to show relative distances for EET from the cytoplasm to external cell environment for each cellular physiology (Hobot et al., 1984; Zuber et al., 2006). Where molecular structure of a protein is used, the protein image was acquired from the Research Collaboratory for Structural Bioinformatics Protein Database (RCSB PDB) and is represented accurately to scale. Cartoon representatives are used to indicate the presence of an enzyme with an unresolved molecular structure, and thus the sizes cannot be drawn to scale. “?” is used to represent uncertainty in the proposed pathway. The inlay in panel **(I)** is used to show the source of electrons and protons coming from NADH oxidation via NADH dehydrogenase located on the cellular membrane of Gram-negative and Gram-positive bacteria (NADH dehydrogenase shown is from *Escherichia coli*, Efremov and Sazanov, 2011). This inlay applies for panel **(I,II)**, and is only shown once to conserve space. Panel **(IA)** represents the PilA pathway in *Geobacter sulfurreducens* that is proposed to use conductive nanowires to perform direct long range EET at distances up to several microns (Reardon and Mueller, 2013; Mulvankar et al., 2015). Panel **(IB)** represents an alternative “OMC” pathway for EET in *G. sulfurreducens* that used a series on redox active proteins which bind directly to an extracellular electron acceptor (Morgado et al., 2011; Zacharoff et al., 2016). Panel **(IC)** represents the Mtr and OmcA pathway(s) for EET in *Shewanella oneidensis* that use a series of redox active proteins which can either bind directly to an extracellular electron acceptor or reduce extracellular flavins which shuttle electrons to an extracellular electron acceptor, resulting in mediated EET (Taylor et al., 1999; Marsili et al., 2008; Firer-Sherwood et al., 2011; Edwards et al., 2014, 2015). The MtrAB complex structure is adjusted from Edwards et al. (2018). The CymA structure is represented by the analogous NrfH from *Desulfovibrio vulgaris* (Rodrigues et al., 2006). Panel **(ID)** represents the production of membrane based nanowires in *S. oneidensis* that use the Mtr and OmcA pathway(s) for direct long range EET at distances up to several microns (Pirbadian et al., 2014; Subramanian et al., 2018). Panel **(IIA)** represents a putative EET pathway that uses a potential combination of lipoteichoic acids [which are bound to the cellular membrane via diacylglycerol (D)] and wall teichoic acids to transfer electron across the peptidoglycan layer (Beveridge and Murray, 1980; Beveridge et al., 1982; Ehrlich, 2008). Panel **(IIB)** represents the MHC pathway from *Thermincola potens* which proposes that EET via direct contact to an extracellular electron acceptor occurs via a series of multiheme cytochromes (Cyts) that are embedded in the peptidoglycan layer, perhaps bound to teichoic acids (Carlson et al., 2012). Panel **(IIC)** shows that the MHC pathway currently does not account for direct long range EET that can transfer electrons at distances up to several microns in *T. ferriacetica* (Parameswaran et al., 2013; Lusk et al., 2016). Panel **(IID)** shows a flavin based model for mediated EET that transfers electrons through the peptidoglycan layer using a series of redox shuttles that may or may not bind to proteins embedded in the peptidoglycan layer (Light et al., 2018; Pankratova et al., 2018). In *Listeria monocytogenes*, this pathway uses a novel NADH dehydrogenase called Ndh2 (Light et al., 2018).

There are three known methods through which exoelectrogens perform EET; with the method employed varying by bacterial species (Torres et al., 2010; Mohan et al., 2014). One method for mediated, or indirect, electron transfer (MET) and two methods for direct electron transfer (DET) (Torres et al., 2010; Mohan et al., 2014). The MET method involves redox mediators, or extracellular shuttles, that are produced by the bacteria and transfer electrons between the bacterial cell and an external metal oxide (Schröder, 2007; Mohan et al., 2014). The two methods for DET to an extracellular electron acceptor include direct contact of a redox protein, often a cytochrome, imbedded on a cell’s outer membrane or peptidoglycan layer to a metal oxide, and long-range (several μm) direct contact of an electrically conductive, or semiconductive, extracellular matrix. The extracellular matrix is hypothesized to confer DET via “nanowires” that are either pili, membranous extensions embedded with redox proteins (often Cyts), or potentially filaments composed entirely of cytochrome monomers (Lovley, 2008; Torres et al., 2010; Carlson et al., 2012; Parameswaran et al., 2013; Pirbadian et al., 2014; Wang et al., 2019). See Figure 1 for an overview of EET in Gram-negative and Gram-positive bacteria.

## Through the Looking Glass

The research concerning the study of EET has focused primarily on Gram-negative mesophilic microorganisms including *Geobacter sulfurreducens* (Bond and Lovley, 2003; Zacharoff et al., 2016), *Geobacter metallireducens* (Lovley, 1993b), and *Shewanella oneidensis* (Lies et al., 2005), with little emphasis on Gram-positive thermophiles. This is a significant distinction since the EET pathways of Gram-negative mesophilic bacteria including *Geobacter* are relatively new adaptations, suggesting that they have a separate evolutionary lineage from those of thermophiles (Holmes et al., 2016). Microbial life on Earth has evolved to produce a bimodal distribution of growth, with the highest growth rates congregating at 42°C and 67°C, implying a potential evolutionary transition around 50°C (Amend and Shock, 2001; Corkrey et al., 2016a,b). In addition, although the genome sizes of thermophiles and mesophiles are roughly equivalent, thermophilic genomes have less intergenic regions and reduced variability in their size (Sabath et al., 2013; Van Noort et al., 2013; Wang et al., 2015). For example, all known microorganisms that thrive at temperatures in excess of 60°C have genomes smaller than 4 megabases (Mb) while all microorganisms with genomes larger than 6 Mb thrive at temperature less than 45°C (Sabath et al., 2013; Van Noort et al., 2013). As a consequence, thermophilic cells tend to have less volume, giving them a higher surface area to volume ratio with their surroundings (Dufresne et al., 2005; Giovannoni et al., 2005; Moya et al., 2009; Dill et al., 2011). Finally, an increase in optimal growth temperature has a large impact on the GC content of the bacterial RNA, affects the amino acid sequence of almost every protein within the thermophilic proteome including the frequency and distribution of amino acid residues in the transmembrane domain, and reduces protein length and family size (Hurst and Merchant, 2001; Saunders et al., 2003; Das et al., 2006; Puigbò et al., 2009; Burra et al., 2010; Dutta and Chaudhuri, 2010; Meruelo et al., 2012; Sabath et al., 2013).

It is well documented that thermophilic enzymes (thermozymes) have higher thermostability than mesophilic enzymes (Vieille and Zeikus, 1996, 2001; Friedman et al., 2004; Li et al., 2005). In addition, thermophiles also have a much lower rate of non-synonymous substitutions in protein coding regions of their DNA than do mesophiles (Friedman et al., 2004; Van Noort et al., 2013; Wang et al., 2015). This is observed when measuring the ratio of non-synonymous substitutions (K_a_) –changes in codon sequence that affect amino acid sequence- to synonymous substitutions (K_s_)–changes in codon sequence that do not affect amino acid sequence (Yang and Bielawski, 2000; Hurst, 2002). The smaller K_a_/K_s_ ratio (ω) (Supplementary Equation S1) observed in thermophiles favors stabilizing selection, implying a strong selective pressure for conservation of thermophilic protein structure and function (Sabath et al., 2013; Wang et al., 2015). The functional stability of thermophilic microorganisms resulting from low non-synonymous mutation rates makes them ideal candidates for conducting studies with high reproducibility and constructing bioreactors for long-term bioremediation (Al-Maghrabi et al., 1999; Chaalal and Islam, 2001; Margesin and Schinner, 2001) or bioenergy production (Bergquist et al., 1987; Patel et al., 2019).

The Gram-positive nature of many thermophiles, coupled with the genomic and proteomic impacts of surviving at high temperature, makes it probable that they use alternative mechanism(s) for EET than those present in Gram-negative mesophilic bacteria. The field of study for exoelectrogens has only a cursory understanding of the mechanisms for EET in Gram-positive thermophiles; however, research suggests that the mechanisms substantially differentiate from mesophilic bacteria. For example, the truncated *pilA* gene which codes for the formation of electrically conductive pili (Richter et al., 2008; Holmes et al., 2016) that transfer electrons in *Geobacter* is not found in Gram-positive thermophiles including *Thermincola potens* (Wrighton et al., 2011; Carlson et al., 2012), *Thermincola ferriacetica* (Lusk et al., 2015a), *Thermoanaerobacter ethanolicus* (Holmes et al., 2016), or *Thermoanaerobacter pseudethanolicus* (Lusk et al., 2015b). In addition, proteomic and genomic data indicate that these microorganisms also lack the *Mtr* (Coursolle et al., 2010) and *Omc* (Leang et al., 2003) gene clusters that code for the production of pathways containing multiheme c-type cytochromes (Cyts) that transfer electrons extracellularly in Gram-negative mesophiles (Bird et al., 2011; Carlson et al., 2012; Vecchia et al., 2014; Lusk et al., 2015a, 2018b; Wang et al., 2019).

Currently, the proposed mechanism for EET in many Gram-positive thermophiles, derived from studies with *T. potens*, is a “MHC” pathway consisting of observed and putative Cyts which transcend the entire cell envelope and bind directly to extracellular oxides (Wrighton et al., 2011; Carlson et al., 2012; Shi et al., 2016). Nevertheless, there is not consensus on the precise mechanism(s) for EET in Gram-positive thermophiles since the MHC hypothesis does not explain the observation of direct long range EET present in some, including *T. ferriacetica*, because it does not consider the transfer of electrons through a conductive or semi-conductive extracellular matrix (Parameswaran et al., 2013; Lusk et al., 2016, 2018b). Finally, the crux of the proposed model for an MHC pathway containing Cyt relies on the presence of Cyts. However, some Gram-positive thermophilic exoelectrogens, including *T. pseudethanolicus*, have been discovered that contain no Cyts (Lusk et al., 2015b).

This genetic data, in concert with the physical limitations and ohmic resistance associated with transferring electrons across a thick peptidoglycan layer and eventually through an extracellular matrix, suggests that the EET present in distinct bacterial phyla are the result of a convergent evolutionary process leading to homoplasy. Considering that prokaryotes have been around for 3.5–4 billion years and survived for ∼1.5–2.0 billion years (Sleep, 2018) before atmospheric oxygen (Partin et al., 2013), convergent EET phenomena in prokaryotes is likely. Furthermore, EET likely evolved with similar selective pressures (i.e., the availability of insoluble metal oxides as electron acceptors) across many bacterial phyla. Thus, despite the fundamentally different genetic and proteomic infrastructures of the extracellular matrices contained by Gram-negative mesophiles and Gram-positive thermophiles, similar EET and proton transport limitations are observed in either bacterial physiology (Torres et al., 2008; Wrighton et al., 2011; Carlson et al., 2012; Lusk et al., 2016, 2018b; Holmes et al., 2016).

## Some Like It Hot: Modeling with Thermophilic Microorganisms

In lieu of the genetic and proteomic differences between Gram-negative mesophiles and Gram-positive thermophiles, the development and verification of mathematical models can be used to enhance our understanding of EET and its limitations, and potentially to devise productive next generation biotechnologies. Obtaining data in order to construct models for EET often proceeds by cultivating dissimilatory metal reducing microbes on electrodes attached to voltammeters or potentiostats in bioelectrochemical systems (BES) or microbial electrochemical cells (MxCs) (Marcus et al., 2007, 2011; Hamelers et al., 2011; Peng et al., 2013; Renslow et al., 2013). Once grown on electrodes, electrochemical techniques including CV, CA, and EIS can be used to monitor and characterize the influence of a Gram-positive thermophilic cellular physiology on the conductivity of the extracellular matrix (K_bio_) and changes in the redox potential (E_KA_) of electron channeling proteins during EET (Srikanth et al., 2008; Marsili et al., 2010; Strycharz et al., 2011; Yang et al., 2012; Badalamenti et al., 2013; Parameswaran et al., 2013; Yoho et al., 2014, 2015; Tan et al., 2017; Lusk et al., 2018b). For example, research using these electrochemical techniques has already shown the presence of multiple redox pathways and proton coupled EET in exoelectrogens, including Gram-positive thermophiles (Badalamenti et al., 2013; Fu et al., 2013; Yoho et al., 2014, 2015; Zacharoff et al., 2016; Lusk et al., 2018b).

The reasons for these phenomena are worth investigating given that metal-like conductance along teichoic acids or electron hopping along cytochromes embedded in peptidoglycan, particularly at high temperatures, ought to have influences on the limitations of EET in Gram-positive thermophiles compared to Gram-negative mesophiles due to changes in K_bio_ and E_KA_ (Marcus et al., 2007; Torres et al., 2010; Yates et al., 2016; Lusk et al., 2018b). In addition, with increasing temperature, the Michaelis–Menten (Supplementary Equation S2) saturation curve predicts a higher substrate concentration at which the reaction rate is half of the maximum (*K_M_*). Nevertheless, proteins produced by thermophiles maintain similar reaction kinetics and binding affinities to those observed in mesophilic organisms (Marcus et al., 2011; Dong et al., 2018). This is because thermophiles maintain a robust repertoire of genes and proteins that provide them with marginal stability via the regulation of ions, organic molecules, and other osmolytes (Marcus et al., 2007; Okur et al., 2017; Lusk et al., 2018b). Thus, despite the case that the redox potential of cytochromes is highly influenced by pH, ohmic resistance, and temperature, thermophiles have similar redox potentials when compared to their mesophilic counterparts (Chang and Kim, 2005; Liu et al., 2011; Wrighton et al., 2011; Carlson et al., 2012; Deng et al., 2013; Pirbadian et al., 2014; Vecchia et al., 2014; Lusk et al., 2018b). As a consequence, currently no mathematical models of EET draw definitive distinctions concerning limitations in EET resulting from either Gram-positive or Gram-negative bacteria (Marcus et al., 2007, 2011; Hamelers et al., 2011; Peng et al., 2013; Renslow et al., 2013).

A common hypothesis assumes that the evolutionary selection for small genomes and smaller, thermally stable proteins in thermophiles minimizes energy expenditures for cell maintenance and protein synthesis (Harold, 1986; Wagner, 2005; Lane and Martin, 2010; Sabath et al., 2013). Although genome replication only accounts for 2% of a microbe’s energy budget, translation of genetic information into proteins is a high energy cost to the cell, accounting for ∼75% of a cell’s energy expenditures (Harold, 1986; Wagner, 2005; Lane and Martin, 2010). Measurements garnered from MxC experiments involving Gram-positive thermophiles have corroborated this hypothesis, showing that thermophilic exoelectrogens have increased growth (μ) (Supplementary Equation S3) and metabolic rates (*r_ut_*) (Supplementary Equation S4) while reducing the fraction of electrons received from electron donors that are used for cell maintenance and synthesis of cellular material (f_s_^°^), resulting in lower biomass production per electron consumed [measured as yield (*Y*)] (Supplementary Equation S5) and a greater number of electrons from the electron donor that can be captured from the biomass (measured as CE) (Supplementary Equation S6) (Makarieva et al., 2005; Lee et al., 2009; Parameswaran et al., 2013; Lusk et al., 2018a).

A consequence of cultivating thermophilic microbes is that increasing temperature has a significant impact on the thermodynamics of electron donor – electron acceptor interactions (Supplementary Table S1). For example, when growing microorganisms in bioreactors, including MxCs, it is vital to add buffer in order to mitigate pH inhibition and proton transport limitations (Torres et al., 2008; Lusk et al., 2016); however, operating at 60°C compared to 30°C impacts the pKa of the buffer added to the system (Supplementary Table S2). For example, in a sodium bicarbonate buffered system, the pKa_1_ drops from 6.33 to 6.30 and the pKa_2_ drops from 10.29 to 10.14 (Supplementary Equation S7) (Mook and Koene, 1975; Lusk et al., 2015c). Furthermore, the potential of the external electron acceptor for exoelectrogens is dependent upon the temperature and the pH of their environment (Supplementary Table S3) (Logan et al., 2006). At 30°C, the change in potential is ∼60 mV per pH unit; however, this change is 66 mV per pH unit at 60°C resulting in about a 1 mV per 5°C change in the energetics of EET (Supplementary Equation S8) (Katuri et al., 2010; Brownson and Banks, 2014; Lusk et al., 2015c, 2018b). In addition, using a simplified Einstein-Stokes equation, the rate for diffusion at 60°C is ∼2 × faster than 30°C (Supplementary Equation S9) (Lusk et al., 2015c). Also, Henry’s Law constants and the Van’t Hoff equation reveal that solubility of O_2_ in water is 35% less at 60°C compared to 30°C, reducing O_2_ contamination issues in anaerobic bioreactors (Supplementary Equation S10) (Lusk et al., 2015c, 2018a). As a result, research with thermophilic bacteria grown as biofilms in MxCs indicates that they may be good candidates for developing next generation biotechnologies since they are able to send more electrons from substrates to extracellular electron acceptors (measured as current density, or *j*) and at lower buffer concentration than are mesophiles (Torres et al., 2008; Parameswaran et al., 2013; Lusk et al., 2016, 2018a).

Nevertheless, there are practical limitations to consider when operating biotechnologies at thermophilic conditions. For example, it is important to mitigate evaporation given that the Antoine equation shows that the vapor pressure for water is ∼4.5 × greater at 60°C compared to 30°C (Supplementary Equation S11) (Rodgers and Hill, 1978). In addition, certain essential gasses are less soluble at 60°C compared to 30°C which may impact microbial growth (Varshney et al., 2016). Carbon dioxide (CO_2_), for example, is essential for the growth of many photosynthetic microbes (Singh and Singh, 2014) and is ∼55% less soluble at 60°C compared to 30°C (Carroll et al., 1991). The high energy input required to maintain thermophilic conditions makes the implementation of thermophiles in biotechnologies practical for applications involving thermophilic liquids, including the removal of organic material (measured as COD or BOD) from compost, brewery, sugarcane, beet sugar, or molasses wastes (Skjelhaugen, 1999; Zupančič et al., 2007; Bhatnagar et al., 2016).

## All in the Family?

More data is needed on the genealogy of EET pathways and the dynamic genomics, transcriptomics, proteomics, and metabolomics of exoelectrogens performing dissimilatory metal reduction (Zhang et al., 2014; Leary et al., 2015; Wang et al., 2015; Eddie et al., 2017). Furthermore, investigating the role of horizontal gene transfer (HGT) from thermophilic archaea to bacteria, which has been shown to make up as much as 24% of the thermophilic bacterial genome, may reveal EET mechanisms that persist across multiple domains of life and thus provide additional insight into the evolutionary history of EET (Aravind et al., 1998; Nelson et al., 1999; Gu and Hilser, 2009; Yilmazel et al., 2018). More information is needed to draw distinctive conclusions regarding the precise mechanisms for EET in exoelectrogens. Nevertheless, understanding the mechanisms and physiology for EET across diverse microbial species will elucidate the path(s) through which selective pressure to reduce extracellular metal oxides may have led to independent evolutionary adaptations.

To acquire a more fundamental understanding of the processes occurring during dissimilatory metal reduction, it is important to recover RNA from the biomass of respiring exoelectrogens for a transcriptomic analysis of the genes that are being transcribed during EET (Qiao et al., 2009). In addition, it is also essential to probe into the proteomic data of these bacteria to gather a more complete understanding of which proteins are being translated and expressed during dissimilatory metal reduction (Vecchia et al., 2014; Costa et al., 2015, 2018). For example, observing upregulation and downregulation of transcribed genes and translated proteins, and production or cessation of metabolites in the presence of varying electron acceptors and donors will elucidate which respiratory pathways are essential for EET in Gram-positive thermophilic and Gram-negative mesophilic exoelectrogens (Zhang et al., 2014; Leary et al., 2015; Wang et al., 2015; Eddie et al., 2017).

Furthermore, given the pivotal role thermophilic exoelectrogens perform in the Earth’s biogeochemical cycles (Wiegel and Michael, 1998; Fenchel et al., 2012; Partin et al., 2013), increasing our understanding of these microorganisms allows us to gain insight into the processes which formed the composition of early Earth and continue to form its composition in the present day, and perhaps present new opportunities for investigating the presence of exobiological phenomenon on distant planets (Bulat et al., 2011; Girguis and Fundis, 2018). Finally, the discovery of novel thermophilic microorganisms containing thermozymes has large economic potential for the development of novel and existing biotechnologies (Brock and Freeze, 1969; Turner et al., 2007; Mathis et al., 2008; Dopson et al., 2016; Lusk et al., 2018a). These factors make the study of Gram-positive thermophilic exoelectrogens, in addition to well documented studies in Gram-negative mesophilic and thermophilic (Fu et al., 2013) exoelectrogens, essential for increasing our understanding of the function and origins for respiration on Earth, optimizing emerging biotechnologies, and creating new solutions to mitigate contamination remediation and energy development.

## Author Contributions

The author confirms being the sole contributor of this work and has approved it for publication.

## Conflict of Interest Statement

The author declares that the research was conducted in the absence of any commercial or financial relationships that could be construed as a potential conflict of interest.

## Abbreviations

BES: bioelectrochemical systems
BOD: biochemical oxygen demand
CA: chronoamperometry
CE: coulombic efficiency
COD: chemical oxygen demand
CV: cyclic voltammetry
Cyt: multiheme c-type cytochrome
D: diacylglycerol
DET: non-mediated direct electron transfer
DMK: demethylmenaquinone
DNA: deoxyribonucleic acid
EET: extracellular electron transfer
EIS: electrochemical impedance spectroscopy
E_KA_: redox potential of respiratory pathway
Fcc_3_: flavocytochrome C3
f_s_^°^: fraction of electrons received from electron donors that are used for cell maintenance and synthesis of cellular material
Ga: gigaannum or one billion years
GC content: guanine and/or cytosine content
HGT: horizontal gene transfer
K_a_: non-synonymous substitutions
K_bio_: conductivity of biomass
*K_M_*: substrate concentration at which the reaction rate is half of the maximum
K_s_: synonymous substitutions
Lpp: lipoprotein
Mb: megabases or million base pairs
MET: mediated or indirect electron transfer
MHC: multiheme cytochrome
mV: millivolts
MxC: microbial electrochemical cell
NAG: N-Acetylglucosamine
NAM: N-Acetylmuramic acid
nm: nanometer
ω: K_a_/K_s_ ratio
pKa: logarithmic acid dissociation constant
Q: quinone
RNA: ribonucleic acid
*r_ut_*: metabolic rate
μ: growth rate
μm: micrometer
*Y*: biomass yield
