# Supplementary material for: Thermophiles; or, the Modern Prometheus: The Importance of Extreme Microorganisms for Understanding and Applying Extracellular Electron Transfer
Source: Front Microbiol. 2019 Apr 26;10:818. doi: 10.3389/fmicb.2019.00818 (PMC6497744; doi:10.3389/fmicb.2019.00818)
Supplement: Supplementary file 1 [file Table_1.docx]

Thermophiles; or, the Modern Prometheus: The Importance of Extreme Microorganisms for Understanding and Applying Extracellular Electron Transfer

Bradley G. Lusk^1^

1. ScienceTheEarth, Mesa, Arizona 85201, United States

SUPPLEMENTAL MATERIAL:

(S1) ω = $\frac{K_{a}}{K_{s}}$

**EQUATION S1:** K_a_/K_s_ ratio (ω). Where K_a_ is equal to the number of nonsynonymous mutations per nonsynonymous site and K_s_ is equal to the number of synonymous mutations per synonymous site. Generally, ω = 1 is neutral selection, ω > 1 is positive/ diversifying selection, and ω < 1 is negative/ purifying selection

(S2)
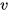
 =
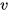
_max_ $\frac{S}{K_{m}+S}$

**EQUATION S2:** Michaelis-Menten equation (
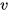
). Where
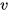
_max_ is equal to the maximum rate, S is equal to the concentration of substrate, and K_m_ is the substrate concentration at which the reaction rate is ½
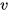
_max_.

(S3) µ = µ_max_ $\frac{S}{K_{s}+S}$

**EQUATION S3:** Monod equation for growth (µ). Where µ_max_ is equal to the maximum growth rate, S is equal to the concentration of substrate, and K_s_ is the substrate concentration at which the reaction rate is ½ µ_max_.

(S4) *r_ut_* = q_max_X_f_ $\frac{S}{K_{s}+S}$

**EQUATION S4:** Derived Monod equation for substrate utilization (*r_ut_*). Where q_max_ is equal to the maximum substrate utilization rate, X_f_ is equal to the biomass concentration, S is the concentration of the rate limiting substrate, and K_s_ is the substrate concentration at which the reaction rate is ½ q_max_.

(S5) *Y* = $\frac{{(f}_{s})(\frac{1}{n_{B}})({MW}_{B})}{(\frac{1}{n_{S}})({MW}_{S})}$ or *Y* = $\frac{quantity of biomass produced}{quantity of substrate consumed}$

**EQUATION S5:** Biomass yield (*Y*). Where *f*_s_ is equal to the fraction of electrons used from the substrate for biomass synthesis, n_B_ is equal to the number of electron equivalents in a mole of biomass, MW_B_ is equal to the molecular weight of the biomass, n_S_ is equal to the number of electron equivalents in a mole of substrate, and MW_S_ is equal to the molecular weight of the substrate.

(S6) CE = $100* \frac{C_{C}}{C_{S}}$

**EQUATION S6:** Columbic efficiency (CE). Where C_C_ is equal to the amount of coulombs captured from the substrate and C_S_ is equal to the amount of coulombs removed from the substrate.

(S7a) pKa_1_ = $\frac{3404.71}{T}$ + 0.032786*T – 14.8435

(S7b) pKa_2_ = $\frac{2902.39}{T}$ + 0.02379*T – 6.4980

**EQUATION S7 a and b:** Temperature dependence of pKa_1_ and pKa_2_ of sodium bicarbonate (Mook and Koene, 1975). Where T is equal to temperature in Kelvin (K).

(S8) *E_acp_* = $E_{acp}^{^{\circ}}$ - $(\frac{RT}{nF}$)(ln $\frac{[{S]}^{a}}{{[P]}^{b}* [H^{+}]^{c}}$)

**EQUATION S8:** Impact of pH on the potential of electron acceptor vs standard hydrogen electrode (SHE) at standard conditions. Where E_acp_ is equal to the theoretical potential of the electron acceptor, R is equal to the ideal gas constant, T is equal to the temperature (K), n is equal to the number of electrons per mole of substrate, S is equal to the concentration of substrate, a is equal to the moles of substrate, P is equal to the concentration of product, b is equal to the moles of product, H^+^ is equal to protons, and c is equal to the moles of protons.

(S9) D_2_ = D_1_ ($\frac{T_{2}x {Vis}_{H_{2}O, 2}}{T_{1}x {Vis}_{H_{2}O, 1}})$

**EQUATION S9:** Simplified Einstein-Stokes equation (*D*). Where D_1_ is equal to the diffusion of an ion at temperature 1, D_2_ is equal to the diffusion of an ion at temperature 2, T_1_ is equal to the temperature (K) at time 1, T_2_ is equal to the temperature (K) at time 2, Vis_H2O, 1_ is equal to the viscosity of H_2_O at T_1_, and Vis_H2O, 2_ is equal to the viscosity of H_2_O at T_2_.

(S10) K_H, cp_ (T) = K_H, cp_ (T^θ^) * $e^{C* \frac{1}{T}-\frac{1}{T^{\theta}}}$

**EQUATION S10:** Van’t Hoff equation. Where K_H,cp_(T) = Henry’s Law constant of O_2_ for a given concentration and pressure (mol /L*atm) at temperature (K), K_H,cp_(T)^θ^ = Henry’s Law constant of O_2_ under standard concentration, pressure and temperature (K), and C = enthalpy of solution at standard temperature/ ideal gas constant.

(S11) log_10_ *P* = A- $\frac{B}{C+T}$

**EQUATION S11:** Antoine equation. Where *P* is equal to the vapor pressure in mmHg and T is equal to the temperature in Celsius (°C). When applying this equation to calculate vapor pressure of H_2_O, A is equal to 8.07131 from 1-99 °C and 8.14019 from 100-374 °C, B is equal to 1730.63 from 1-99 °C and 1810.94 from 100-374 °C, and C is equal to 233.426 from 1-99 °C and 244.458 from 100-374 °C (Rodgers and Hill, 1978).

| (a) | E_acp_ (V vs SHE) | E_don_ (v vs SHE) | $\Delta$E (V vs SHE) |
| --- | --- | --- | --- |
| Standard (E^0^)^1^ | 1.229 | 0.187 | 1.042 |
| 25°C^2^ | 0.805 | -0.296 | 1.101 |
| 30°C | 0.797 | -0.305 | 1.102 |
| 60°C | 0.754 | -0.353 | 1.107 |

| (b) | E_acp_ (V vs SHE) | E_don_ (V vs SHE) | $\Delta$E (V vs SHE) |
| --- | --- | --- | --- |
| Standard (E^0^)^1^ | 0.0 | 0.187 | -0.187 |
| 25°C^2^ | -0.414 | -0.296 | -0.117 |
| 30°C | -0.420 | -0.305 | -0.115 |
| 60°C | -0.462 | -0.353 | -0.109 |

**Table S1a and b:** The impact of temperature on the thermodynamics between the electron donor and acceptor. Table S1a shows that the theoretical potential of the electron donor (5 mM acetate) (E_don_) and the electron acceptor (oxygen at a partial pressure of 0.2 atmospheres (atm)) (E_acp_) shift more negative as temperature is increased. As a result, the theoretical potential difference ($\Delta$E) between the electron donor and acceptor (displayed assuming 5 mM bicarbonate at pH 7 in comparison to SHE) increases slightly as temperature is increased (Lusk et al., 2015). This may be beneficial for operating microbial fuel cells (MFCs) since similar or greater current (*j* or Amps) carried over a greater potential gradient (Volts) will produce more power (Watts). Table S1b shows that the theoretical potential of the electron donor (5 mM acetate) (E_don_) and the electron acceptor (H_2_O during catalysis for H_2_ generation at a partial pressure of 1 atm). As a result, the theoretical potential difference ($\Delta$E) between the electron donor and acceptor (displayed assuming 5 mM bicarbonate at pH 7 in comparison to SHE) decreases slightly as temperature is increased (Lusk et al., 2015). This may be beneficial for operating microbial electrolysis cells (MECs) since it implies that lower voltage inputs are required to drive the electrolysis of H_2_O during cathodic H_2_ production. ^1^Data from (Thauer et al., 1977) and ^2^data from (Logan et al., 2006). Table adapted from (Lusk et al., 2015).

| T (°C) | pKa_1_ | pKa_2_ |
| --- | --- | --- |
| 0 | 6.58 | 10.63 |
| 25 | 6.35 | 10.33 |
| 30 | 6.33 | 10.29 |
| 60 | 6.30 | 10.14 |

**Table S2:** Impact of temperature on pKa of bicarbonate buffer. Table shows a theoretical decrease in pKa_1_ and pKa_2_ of sodium bicarbonate with increasing temperature. Table adapted from (Lusk et al., 2015).

| T (°C) | ∆mV per pH unit |
| --- | --- |
| 0 | 54.2 |
| 25 | 59.1 |
| 30 | 60.1 |
| 60 | 66.1 |

**Table S3:** Impact of temperature on thermodynamics of electron acceptor. Table shows a theoretical ~1.0 mV change in electron acceptor potential per 5.0 °C. Table adapted from (Lusk et al., 2015).

**REFERENCES**

Some sections and ideas in this manuscript were first discussed in the author’s dissertation titled *Thermophilic Microbial Electrochemical Cells*.

Logan, BE, Hamelers, B, Rozendal, R, Schroder, U. 2006. Microbial Fuel Cells: Methodology and Technology. Environ. Sci. Technol. 40:5181.

Lusk, B.G., Torres, C.I., Krajmalnik-Brown, R., Rittmann, B.E. 2015. Thermophilic Microbial Electrochemical Cells, [Dissertations Theses]. [Tempe (AZ)]; Arizona State University.

Mook, WG, Koene, BKS. 1975. Chemistry of dissolved inorganic carbon in estuarine and coastal brackish waters. Estuarine and Coastal Marine Science. 3:325-336.

Rodgers, R., Hill, G. 1978. Equations for vapour pressure versus temperature: Derivation and use of the Antoine equation on a hand-held programmable calculator. British Journal of Anaesthesia, 50(5), 415-24.

Thauer, RK, Jungermann, K, Decker, K. 1977. Energy conservation in chemotrophic anaerobic bacteria. Bacteriol. Rev. 41:100-180.
